# Polymorphisms in *TNF Receptor Superfamily 1B* (*TNFRSF1B:rs3397*) are Linked to *Mycobacterium avium paratuberculosis* Infection and Osteoporosis in Rheumatoid Arthritis

**DOI:** 10.3390/microorganisms7120646

**Published:** 2019-12-04

**Authors:** Amna Naser, Ahmad K. Odeh, Robert C. Sharp, Ahmad Qasem, Shazia Beg, Saleh A. Naser

**Affiliations:** 1 Department of Anatomy and Cell Biology, Brody School of Medicine. East Carolina University, Greenville, NC 27858, USA; Amna.naser@knights.ucf.edu; 2 Division of Molecular Microbiology, Burnett School of Biomedical Sciences, College of Medicine, University of Central Florida, Orlando, FL 32816, USA; ahmadkodeh@Knights.ucf.edu (A.K.O.); ahmadqasem@knights.ucf.edu (A.Q.); 3 Department of Pathology, Immunology and Laboratory Medicine, College of Medicine, University of Florida, Gainesville, FL 32611, USA; rsharp07@ufl.edu; 4 College of Medicine, UCF Health, University of Central Florida, Orlando, FL 32827, USA; Shazia.beg@ucf.edu

**Keywords:** rheumatoid arthritis, Mycobacterium paratuberculosis, osteocalcin, osteoporosis, MAP, RA

## Abstract

We previously discovered that single nucleotide polymorphisms (SNPs) in *PTPN2/22* (T-cell negative-regulators) occur in 78% of rheumatoid arthritis (RA), along with *Mycobacterium avium paratuberculosis* (MAP) infection in 33% of patients. In Crohn’s disease, we reported that SNPs in *TNFα* and receptors (*TNFRSF1A/TNFRSF1B*) benefited intracellular MAP-survival, increased infection, and elevated inflammatory response mimicking the poor response to anti-TNF*α* treatment in some patients. Here, we studied the frequency and effects of SNPs in *TNFα/TNFRSF1A/TNFRSF1B* in RA including gene expression, MAP infection, and osteoporosis marker levels in blood (54 RA and 48 healthy controls). *TNFα:rs1800629* (GA) was detected in 19/48 (40%) RA and 8/54 (15%) controls (*p*-value < 0.05, odds ratio (OR) = 3.6, 95% CI: 1.37–9.54). *TNFRS1B:rs3397* (CT) was detected in 21/48 (44%) RA and 10/54 (19%) controls (*p*-value < 0.05, OR = 4.43, 95% CI: 1.73–11.33). In RA, *rs3397* downregulated *TNFRSF1B* expression (CC > CT (0.34 ± 0.14) and CC > TT (0.27 ± 0.12)), compared to wildtype CC (0.51 ± 0.17), *p*-value < 0.05. MAP DNA was detected significantly in 17/48 (35.4%) RA compared to 11/54 (20.4%) controls (*p*-value < 0.05, OR = 2.14, 95% CI: 1.12–5.20). The average osteocalcin level was significantly lower (*p*-value < 0.05) in RA (2.70 ± 0.87 ng/mL), RA + MAP (0.60 ± 0.31 ng/mL), RA + *TNFRSF1B:rs3397* (TT) (0.67 ± 0.35 ng/mL), compared to the healthy control (5.31 ± 1.39 ng/mL), and MAP-free RA (3.85 ± 1.31 ng/mL). Overall, *rs3397* appears to downregulate *TNFRSF1B*, increase MAP infection, worsen inflammation, and cause osteocalcin deficiency and possibly osteoporosis in RA.

## 1. Introduction

Rheumatoid arthritis (RA) is a chronic inflammatory disorder which affects the joints and results in tearing of articular cartilage [1]. Patients suffering from RA are also characterized by having lower bone mineral density, and higher risk of fractures [1,2,3,4,5]. These symptoms most often lead to osteoporosis, which has been linked to vitamin D deficiency and prolonged exposure to corticosteroids treatment [6,7].

The elevated level of pro-inflammatory cytokines, mainly tumor necrosis factor alpha (TNFα), in RA blood has negative effects on osteoclast paracrine stimulation, which leads to bone resorption as seen in osteoporosis [8]. Several studies have shown that increasing osteoclastic activity plays a major role in the development of osteoporosis in RA. For example, Gough et al. reported that bone resorption biomarkers were elevated in RA patients who were suffering from low bone mineral density, which resulted in further evidence that linked osteoclast activity with bone resorption in RA [9]. Although the underlining cause of bone resorption abnormality is still debated, genetic factors such as single nucleotide polymorphisms (SNPs) and environmental triggers are most likely involved in the disease process [10]. Most recently, we reported that the bone biomarker osteocalcin level was low in Crohn’s disease (CD), an inflammatory bowel disease with established association with genetic predisposition and environmental triggers such as *Mycobacterium avium* subspecies *paratuberculosis* (MAP) [10]. Specifically, we observed that the blood levels of active osteocalcin was reduced, whereas the undercarboxylated osteocalcin (ucOC) level was elevated in CD, compared to healthy individuals [10]. Most importantly, the significant decrease in the osteocalcin level in CD blood was associated with MAP infection in these patients. These interesting data suggest a possible role for the combined inflammatory status and MAP infection and the development of osteoporosis in CD patients with key mutations. We further investigated such correlation and we reported that:

SNPs in TNF Receptor Superfamily Member 1A/1B (*TNFRSF1A* and *TNFRSF1B*) have downregulated the expression of their corresponding genes significantly, which resulted in increased susceptibility to MAP infection in CD [11].

Most recently, we discovered that MAP infection also occurred in RA patients who are associated with SNPs in *PTPN2/22*, T-cell negative regulators [12]. Patients with these SNPs exhibit hyper-proliferative T-cells, which suggests a continued hyper inflammatory response in those with MAP infection. The combination of MAP infection, known to induce production of TNFα, and presence of defective T-cells in RA should lead to excessive elevation of inflammatory markers, which ultimately should impact osteoclastic activity and possibly development of osteoporosis [12]. In this study, we focused on investigating the interaction between SNPs in *TNFα* and its receptors (*TNFRSF1A* and *TNFRSF1B*), MAP infection, and active osteocalcin levels (an osteoporosis marker) in blood samples from RA patients.

## 2. Materials and Methods

### 2.1. Clinical Samples

Peripheral blood (two 4.0 mL K_2_-EDTA) from 102 study participants (54 RA patients and 48 healthy controls) were acquired from the University of Central Florida Health Center. All participants provided written informed consent prior to enrollment, and the study was approved by the University of Central Florida Institutional Review Board #SBE-16-12193 (approved on September 19th, 2017). RA diagnosis was performed and reported by Dr. Shazia Beg (University of Central Florida Health) based on approved standard American College of Rheumatology (ACR) criteria for RA diagnosis. This includes a physical exam, imaging and laboratory tests (rheumatoid factor, C-reactive protein (CRP), erythrocyte sedimentation rate (ESR), and anti-cyclic citrullinated peptide (AntiCCP)).

From each participant, one blood tube was processed for the presence of MAP infection, while the second tube was used for gene expression analysis and genotyping of three SNPs in *TNFα*, *TNFRSF1A*, and *TNFRSF1B*. The demographics for all participating subjects are listed in Table 1.

### 2.2. Detection of MAP Infection in the Blood of RA Patients by nPCR

Purified white blood cells separated from the blood samples were analyzed for the presence of MAP DNA. Genomic DNA extraction and nested PCR (nPCR) analysis were performed as described earlier [13,14]. Briefly, nPCR was based on *MAP-specific IS900* derived oligonucleotide primers. The first round of amplification was performed using P90 (GTTCGGGGCCGTCGCTTAGG) and P91 (GAGGTCGATCGCCCACGTGA) primers at the following conditions: 95 °C for 5 min, then 34 cycles of 95 °C for 1 min, 58 °C for 1.5 min, 72 °C for 1.5 min. Final extension of 10 min at 72 °C. The product size amplified from this round was 398 bp. A second round of amplification was followed, which involved using AV1 (ATGTGGTTGCTGTGTTGGATGG) and AV2 (CCGCCGCAATCAACTCCAG) primers at the following conditions: 95 °C for 5 min, then 34 cycles of 95 °C for 1 min, 60 °C for 0.5 min, 72 °C for 1.5 min. Final extension of 10 min at 72 °C. The final PCR amplified fragment was 298 bp and was analyzed on 2% agarose gel.

### 2.3. Measurement of Active Osteocalcin Level

An ELISA sandwich assay was used to measure the serum active osteocalcin level. A Life Technologies Osteocalcin Human ELISA Kit (Life Technologies, Carlsbad, CA, USA) was used following the manufacturer’s instructions. The absorbance readings were measured at 450 nm wavelength and osteocalcin concentration was calculated from the generated standard curve equation following the manufacturer’s instructions.

### 2.4. Identification of SNPs in TNFα, TNFRSF1A, and TNFRSF1B

Extraction and purification of genomic DNA for genotyping was performed using QIAamp DNA Blood Mini Kit (Qiagen, Germantown, MD, USA) following the manufacturer’s instructions. TaqMan genotyping assays (Applied Biosystems, Foster City, CA, USA) for *TNFα:rs1800629*, *TNFRSF1A:rs767455*, and *TNFRSF1B:rs3397* were performed on purified DNA samples following the manufacturer’s protocol at the University of Florida Pharmacotherapy and Translational Research Department (Gainesville, FL, USA). Two solutions; 2 × TaqMan master mix and 20 × assay working stock (primers with VIC and FAM fluorophore attachment) made up the reaction mixture. Following reverse transcription polymerase chain reaction (RT-PCR) assay (one cycle at 95 °C for 10 min, 92 °C for 15 s, and 50 cycles at 58 °C for 1 min), isolated DNA samples in addition to the reaction mixture and controls were added into a 384-well microtiter plate using the Applied Biosystems QuantStudio RT-PCR System. The plate was analyzed for VIC and FAM fluorophores at 551 and 517 nm, respectively. Fluorescence of VIC or FAM alone determined that the sample had allele 1 or allele 2, while VIC and FAM together determined that the sample was heterozygous for each allele. Each selected SNP is described in Table 2.

### 2.5. RNA Extraction and Measurement of TNFα, TNFRSF1A, and TNFRSF1B Expression

Leukocytes from 1.0 mL of blood were isolated in a 2.0 mL RNase free microcentrifuge tube, suspended in 1.0 mL of TRIzol^®^ reagent (Invitrogen) and then subjected to RNA extraction as described earlier [11]. RNA pellets were air-dried for 15–30 min, suspended in 20 μL of RNase free H_2_O, and finally heated at 60 °C for 10 min. Then, 600 ng of RNA from each sample was added to 0.25 mL tubes containing 0.2 mL of PCR reaction, 4 μL of iScript™ Reverse Transcription (Bio-Rad^®^), and up to 20 μL RNase free H_2_O for cDNA synthesis. All tubes were then transferred to a thermal cycler (MyGene™ Series Peltier Thermal Cycler) and run for 5 min at 25 °C, 20 min at 46 °C, and 1 min at 95 °C. The final concentration of cDNA synthesized for each sample was 30 ng/μL. A total volume of 1 μL of cDNA, 10 μL of Fast SYBR Green Mastermix (Thermo Fisher Scientific^®^), 1 μL of either *TNFα*, *TNFRSF1A*, or *TNFRSF1B* PrimePCR SYBER Green Assay mix (Bio-Rad^®^), and 8 μL of molecular biological grade sterile H_2_O were mixed in a 96-well microamp RT-PCR reaction plate. Then, 18s RNA gene oligonucleotide primer controls (forward primer: 5′-GTA ACC CGT TGA ACC CCA TT-3′; reverse primer: 5′-CCA TCC AAT CGG TAG CG-3′) were used in order to acquire baseline CT readings. The RT-PCR reaction was performed using the 7500 Fast Real-Time PCR System (Applied Biosystems^®^). The equation (2^ ^(−∆CT)^ × 1000), where ∆CT = Sample RT-PCR CT reading-18s CT baseline, was used to calculate relative mRNA expression levels.

### 2.6. Statistical Analysis

All statistical tests were performed using GraphPad Prism^®^ 8.02. For the osteocalcin level, data are presented as mean ± SD, where a two-tailed *t* test was used to compare the level in RA patients vs. healthy controls and the presence vs. the absence of MAP infection. For genotype frequency, a two-tailed Z test and odds ratio analysis were used to compare SNPs in RA patients vs. healthy controls. At each gene examined, SNP genotypes were categorized into four groups (major, heterozygous, homozygous, and both heterozygous + homozygous), then each genotype group was tested for significance within its subcategory at *p* < 0.05 and a 95% confidence interval (CI). For gene expression analysis, we compared the average gene expression in RA vs. healthy control for each gene regardless of their genotype using an unpaired two-tailed t-test at *p* < 0.05 and a 95% confidence interval (CI), and then we compared individuals who carried two major alleles with others for each SNP tested using one-way ANOVA, where a Newman–Keuls post-hoc test was selected for multiple comparisons. For MAP infection susceptibility, infection proportions between SNP genotypes and major alleles in the RA group was compared to the healthy control group separately using a two-tailed Z test at *p* < 0.05. Age and gender were not included as covariates, as for all data sets no age or gender effects were observed.

## 3. Results

### 3.1. MAP DNA Detected in RA Patients

Overall, MAP DNA was detected in the blood of 28 out of 102 (27.5%) of participating subjects. Specifically, MAP DNA was detected in 17/48 (35.4%) RA patients and 11/54 (20.4 %) healthy controls (*p*-value < 0.05, odds ratio (OR) = 2.14, 95% CI: 1.12–5.20).

### 3.2. Lower Active Osteocalcin Levels in RA Patients

**Figure 1:**
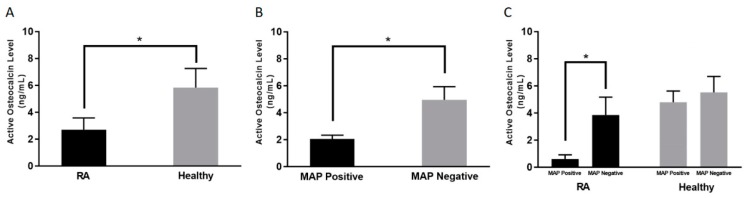
Average active osteocalcin levels among; Rheumatoid Arthritis (RA) patients and healthy controls (**A**), MAP positive and MAP negative regardless of disease condition (**B**), RA and healthy controls in the presence and absence of MAP infection (**C**). * indicates *p* < 0.05.

As shown in Figure 1A, the average active osteocalcin levels in RA patients was 2.70 ± 0.87 ng/mL, compared to 5.31 ± 1.39 ng/mL in healthy controls (*p*-value < 0.05).

### 3.3. Lower Active Osteocalcin Level is Detected in MAP Positive RA Patients

As shown in Figure 1B, regardless of diagnosis, the overall average level of active osteocalcin in the blood samples that have been determined to contain MAP DNA was 2.05 ± 0.24 ng/mL compared to 4.94 ± 0.98 ng/mL (*p*-value < 0.05) in blood samples that are MAP negative. Specifically, the average osteocalcin level in RA patients who are positive for MAP was 0.60 ± 0.31 ng/mL compared to 3.85 ± 1.31 ng/mL (*p*-value < 0.05) in RA patients who are negative for MAP DNA, as shown in Figure 1C.

### 3.4. Frequency of SNPs in TNFα, TNFRSF1A, and TNFRSF1B in RA Patients

We have assessed 102 subjects (48 RA patients and 54 healthy controls) for one SNP in *TNFα* (*rs1800629*)**, two SNPs in *TNFRSF1A* (*rs767455* and *rs4149570*), and one SNP in *TNFRSF1B* (*rs3397*). Genotype distribution of these SNPs conveyed the Hardy–Weinberg equilibrium and are all listed in Table 3. *TNFα:rs1800629* (GG to GA) was significant in RA patients compared to healthy controls (*p*-value < 0.05). Specifically, *TNFα:rs1800629* was detected in 19/48 RA patients (40%) compared to 8/54 healthy controls (15%) (OR = 3.6, 95% CI: 1.37–9.54). *TNFRSF1B:rs3397* (CC to CT) was detected in 21/48 RA patients (44%) compared to 10/54 (10%) healthy controls (OR = 4.43, 95% CI: 1.73–11.33). *TNFRSF1A:rs767455* (AA to AG or GG) was not significant in RA patients.

### 3.5. rs3397 Downregulates TNFRSF1B Expression in RA Patients

**Figure 2:**
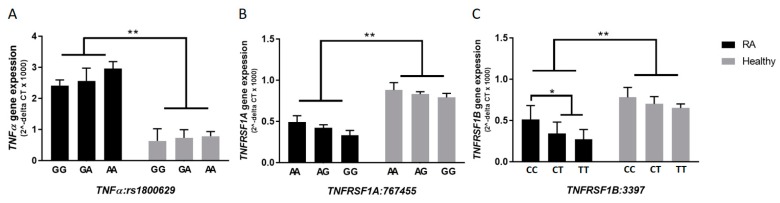
Gene expression levels of *TNFα*, *TNFRSF1A*, and *TNFRSF1B* according to each allele type in selected SNPs (**A**–**C**), among RA patients and healthy controls. * indicates *p* < 0.05, ** indicates *p* < 0.01.

In general, and regardless of the presence or absence of SNPs, the average relative gene expression for *TNFα* was 3-fold higher in RA patients (2.68 ± 0.34), compared to healthy subjects (0.79 ± 0.23) (*p*-value < 0.05). As shown in Figure 2B,C, the relative gene expression of *TNFRSF1A* was 0.42 ± 0.11 in RA compared to 0.81 ± 0.14 in healthy controls (*p-value < 0.05*). The relative gene expression of *TNFRSF1B* was 0.39 ± 0.15 in RA compared to 0.71 ± 0.13 in healthy controls (*p-value < 0.05*). Most importantly, the average relative gene expression level of *TNFRSF1B* was significantly lower in RA patients with *rs3397* (CT: 0.34 ± 0.14 or TT: 0.27 ± 0.12) compared to RA patients with the major genotype (CC: 0.51 ± 0.17) (*p-value < 0.05*), as shown in Figure 2C. On the other hand, *rs767455* did not have any significant effect on *TNFRSF1A* expression, as shown in Figure 2B, and the effect of *rs1800629* on *TNFα* expression was not significant, as shown in Figure 2A.

### 3.6. TNFRSF1B:rs3397 is Associated with Lower Active Osteocalcin Level in RA Patients

**Figure 3:**
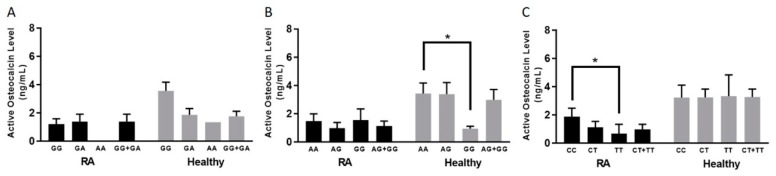
Average active osteocalcin levels based on each allele type of RA patients and healthy controls in selected SNPs: rs1800629 (**A**), rs767455 (**B**), and rs3397 (**C**). * indicates *p* < 0.05.

Among RA patients, only *TNFRSF1B:rs3397* was associated with a lower active osteocalcin level. Blood samples from patients with the CC genotype had a significantly higher level of active osteocalcin (1.86 ± 0.61 ng/mL) (*p*-value < 0.05), compared to samples from patients who had the *TNFRSF1B:rs3397* homozygous (TT) genotype (0.67 ± 0.35 ng/mL), as shown in Figure 3C.

The average active osteocalcin level of healthy controls without the *TNFα:rs1800629* SNP (GG genotype) was 3.55 ± 0.61 ng/mL, compared to the heterozygous or homozygous SNPs (GA or AA genotype), which was 1.75 ± 0.36 ng/mL, as shown in Figure 3A. Similarly, we found that healthy controls who did not have SNP in *TNFRSF1A:rs767455* (AA genotype) had an average active osteocalcin level of 3.43 ± 0.74 ng/mL, which is significantly higher than healthy controls who had a homozygous SNP (GG genotype), which was 0.94 ± 0.17 ng/mL (*p*-value < 0.05), as shown in Figure 3B.

### 3.7. TNFRSF1B:rs3397 is Associated with MAP Infection in RA

**Figure 4:**
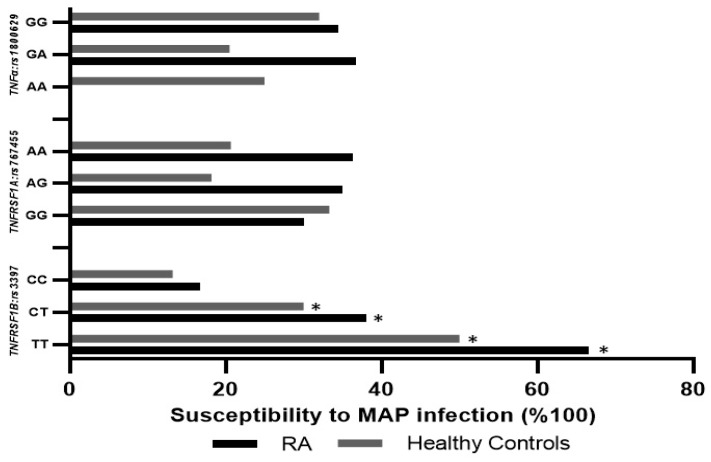
Influence of *TNFα:rs1800629*, *TNFRSF1A:rs767455*, and *TNFRSF1B:rs3397* SNPs on susceptibility to MAP infection in RA patients and healthy controls. * indicates *p* < 0.05.

Blood samples from RA patients with *TNFRSF1B:rs3397* (CT or TT genotype) were significantly positive for MAP DNA (38.1% and 66.6%, respectively), compared to blood from RA patients with the wildtype (CC) genotype (16.7%, *p*-value < 0.05). Similarly, MAP DNA was detected in the blood samples of 50% of the healthy controls with the *TNFRSF1B:rs3397* (TT) genotype, compared to the wildtype (CC) genotype of healthy controls (13.2%, *p-value <* 0.05), as shown in Figure 4. The detection of MAP DNA was not significant in blood samples with *TNFα:rs1800629* or *TNFRSF1A:rs767455*. Additionally, we categorized RA patients into four groups depending on the treatment they were receiving. There was no significant association between treatment option and MAP infection or osteocalcin level, as shown in Table 4.

## 4. Discussion

The development of osteoporosis and osteoporotic fractures are common among RA patients [1,2,3,4,5]. Osteoporosis is associated with a higher level of bone resorption due to inflammation, use of glucocorticoids, and limited physical activity [16,17,18]. Although the mechanism of osteoporosis development in RA patients is still unclear, recent studies have shown that osteoclasts play a major role in the bone erosion process in patients with osteoporosis [19,20,21,22]. Osteoclast’s function is regulated by a balance between TNFα superfamily molecules (such as osteoprotegerin (OPG)), the receptor activator of nuclear factor κB ligand (RANKL), and TNF-related apoptosis-inducing ligand (TRAIL) [23,24]. The mechanisms involved in these interactions and cell signaling have not been elucidated.

Our group has recently established that in vitro infection of macrophages with MAP caused more production of pro-inflammatory cytokines such as TNFα, IL-6, and IL-12 [25]. Several studies have used gene expression profiling to demonstrate the variable response to mycobacterial infections in vitro [26,27,28]. In MAP infected macrophages, expression of quiescent genes (Ephrin-A5, IL-1RA, and IL-7Rα) was higher in comparison to other bacterial species, such as *Mycobacterium avium* subspecies *avium* (MAA) [29]. Additionally, MAA and MAP have substantial effects on expression of apoptosis-related genes, such as FasL and BCL10 [29]. In contrast, MAP infection led to lower expression of major MAPK pathways, including BMP7 and CDK2 [29]. Interestingly, treatment of MAP-infected macrophages with anti-TNFα biologics increased MAP survival and burden, and led to excessive inflammatory response [25]. This is consistent with other studies, which reported a rise in *M. tuberculosis* infection in patients on anti-TNFα treatment [30]. In this study, we confirmed the presence of MAP infection in the blood of RA patients; the majority of these patients were on anti-TNFα treatment. The detection rate of MAP DNA was significantly higher in RA samples compared to healthy controls. This is consistent with our earlier report where MAP was detected in 34% of RA patients [12].

Since MAP was associated with low active osteocalcin levels in CD in an earlier study [10], we were able to confirm this observation in RA. The data in this study showed an association between low serum levels of osteocalcin and MAP infection, regardless of disease status, as shown in Figure 1B. This was further confirmed when the serum level of osteocalcin was lower in blood samples from MAP positive controls (patients who appear to be free from CD or RA), indicating that infection by intracellular pathogens such as MAP directly impact the osteocalcin production mechanism. A low osteocalcin level is one of the indicators of osteoporosis. This raises a point for discussion, which requires further research to confirm or exclude any association between infection, osteocalcin production, and possibly osteoporosis in CD and/or RA.

The shared similarities between CD and RA, especially with regards to TNF*α* profiling and anti-TNF*α* treatment options [31,32], have encouraged us to investigate the effects of these treatment options on increased risk of MAP or *M. tuberculosis* infection and subsequent impact on bone erosions and osteoporosis in these patients. As observed in CD, we determined that the relative expression level of *TNF*α was 2.7-fold higher, while the *TNFRSF1A* and *TNFRSF1B* expression level was 1-fold lower in RA patients compared to healthy controls. More importantly, both the CT and TT genotypes of *TNFRSF1B:rs3397* have significantly reduced gene expression. Coincidentally, MAP was detected significantly higher in the blood of RA patients with *TNFRSF1B:rs3397* compared to wildtype RA. The data strongly associates *TNFRSF1B:rs3397* with MAP infection and low osteocalcin levels in RA. This confirms earlier findings in CD with unique SNPs in *TNFα* receptors (*TNFRS1A:rs767455* and *TNFRS1B:rs3397*) [11].

A critical point to consider in the intersection of immune response and apoptosis is the expression of *TNFRSF1A* and *TNFRSF1B* in MAP-infected cells. MAP infection resulted in lower expression of *TNFRSF1A*, while no changes in expression of *TNFRSF1B* were observed in infected macrophages [33]. Interestingly, *TNFRSF1A* has been recognized as the more pro-apoptotic gene, while *TNFRSF1B* has been linked to apoptosis suppression [34]. *TNFRSF1A:rs767455* and *TNFRSF1B:rs3397* altered expression of corresponding genes, leading to further downregulation, which may cause MAP-infected macrophages to persist with apoptosis and production of higher levels of TNFα. An elevated TNFα level plays a crucial role in the osteoclast paracrine stimulation, which influences bone resorption ultimately [8]. The data clearly demonstrated that dysregulated pro-inflammatory cytokine levels due to persistent MAP infection may result in hyperactive osteoclast activity and alter osteocalcin levels, while the SNPs affected gene expression levels.

Further genetic and functional analyses should be done in order to establish whether these SNPs cause linkage disequilibrium with a nearby-localized gene responsible for the observed effect. It is worth mentioning that TNFα:rs1800629 was strongly associated with the resolution of hepatitis B virus (HBV) infection and reported in susceptibility to human papillomavirus (HPV) infection [35]. The SNP TNFRSF1A:rs767455 was associated with susceptibility to invasive pulmonary aspergillosis (IPA) and TNFRSF1B:rs3397 was reported in occult HBV infection in inflammatory arthritis patients [36,37].

Overall, MAP infection has significantly lowered sera levels of active osteocalcin, a major biomarker for osteoporosis in both CD and RA patients, which confirms our earlier data, where MAP infection decreased active osteocalcin levels in patients with CD [10]. MAP infection was more prevalent in RA patients exhibiting SNPs in *TNFα* receptors (*TNFRS1A:rs767455* and *TNFRS1B:rs3397*). Collectively, the data supports a link between intracellular infection with MAP, SNPs in *TNFα* receptors, and active osteocalcin levels. The conclusion of this study should provoke scientists to consider inflammatory disorders and their complications as an interplay between genetic predisposition, effects of standard treatment options, and acquired infection. A personalized treatment option should be more effective to achieve a cure.

## Figures and Tables

**Table 1:** Demographics of study participants.

Diagnosis	Number	Age Range	Average Age	Gender Ratio (Male/Female %)
All Subjects	102	21–75	39	33:67
Rheumatoid Arthritis	54	22–75	49	25:75
Healthy Controls	48	20–63	30	41:59

**Table 2:** Gene mutations, locations, and mutation phenotypes of single nucleotide polymorphisms (SNPs) selected for this study.

Gene	Reference SNP	Gene Mutation *	Location and AA Change *	Mutation Phenotype
*TNF*	rs1800629	G > A	Promoter	Higher susceptibility to develop CD
*TNFRSF1A*	rs767455	C > T	Exon 4 (R > Q)	Poor response to anti-TNFα treatment in CD
*TNFRSF1B*	rs3397	C > T	Exon 10 (N/A)	Higher susceptibility to MAP infection in CD

* Gene mutation and location data were obtained from the National Center for Biotechnology Information (NCBI) [15]. (R: arginine; Q: glutamine; MAP: Mycobacterium avium subspecies paratuberculosis; CD: Crohn’s disease; MS: multiple sclerosis; N/A: unknown residue change).

**Table 3:** Genotype frequencies of selected SNPs for RA patients and healthy controls.

Genotype	RA Patients (*n* = 48)	Healthy Controls (*n* = 54)	*p*-value *	OR	95% CI
*TNFα:rs1800629*					
GG (Reference allele)	29 (60%)	44 (82%)	0.03		
GA	19 (40%)	8 (15%)	0.02	3.6	1.37–9.54
AA	0 (0%)	2 (0%)	0.23	0.2	0.01–3.36
GA + AA	19 (40%)	10 (19%)	0.04	2.9	1.17–7.07
*TNFRSF1A:rs767455*					
AA (Reference allele)	22 (46%)	29 (54%)	0.42		
AG	20 (42%)	22 (41%)	0.47	1.2	0.53–2.73
GG	6 (12%)	3 (6%)	0.17	2.6	0.59–11.7
AG + GG	26 (54%)	25 (46%)	0.31	1.4	0.63–2.99
*TNFRSF1B:rs3397*					
CC (Reference allele)	18 (37%)	38 (70%)	0.02		
CT	21 (44%)	10 (19%)	0.03	4.43	1.73–11.3
TT	9 (19%)	6 (11%)	0.29	3.17	0.98–10.3
CT + TT	30 (62%)	16 (30%)	0.03	4	1.73–9.05

Two-tailed Z test and odds ratio analysis were used to compare the presence of SNPs in CD patients vs. healthy controls. * *p*-value of *<* 0.05 was considered as the significance threshold. (RA: rheumatoid arthritis; SNP: single nucleotide polymorphism; OR: odds ratio (crude); CI: confidence interval).

**Table 4:** Average osteocalcin levels and MAP infection according to treatment groups among RA patients.

Treatment Group	Number of Patients Receiving Treatment	MAP Infection	Average Osteocalcin (ng/mL)
Anti-TNFα	20	6/20 (30.0%)	2.71 ± 0.82
Methotrexate	28	10/28 (35.7%)	2.49 ± 0.57
Corticosteroids	13	3/13 (23.1%)	2.98 ± 0.69
Hydroxychloroquine	9	4/9 (44.4%)	2.23 ± 0.26
